# Prevention of Work Absence Due to Back Pain: A Network Meta-Analysis

**DOI:** 10.3390/ejihpe13120200

**Published:** 2023-12-10

**Authors:** Kim-Ngan Thi Ta, Chyi-Huey Bai, Kai-Jen Chuang

**Affiliations:** 1 Faculty of Public Health, University of Medicine and Pharmacy at Ho Chi Minh City, Ho Chi Minh City 700000, Vietnam; 2 School of Public Health, College of Public Health, Taipei Medical University, New Taipei City 235, Taiwan; baich@tmu.edu.tw; 3 Department of Public Health, School of Medicine, College of Medicine, Taipei Medical University, New Taipei City 235, Taiwan

**Keywords:** prevention, back pain, backache, work absences, productivity, sickness absenteeism

## Abstract

This paper reviewed the most effective strategies for preventing work absence due to back pain (BP) and BP episodes (the number of people reporting back pain). We searched randomized controlled trials (RCTs) of prevention strategies for BP from previous meta-analyses, PubMed, CENTRAL, and Embase and conducted a network meta-analysis. Thirteen RCTs (2033 participants) were included. Low- to high-quality evidence showed that exercise combined with ergonomics, education, back belts, and education combined with ergonomics did not prevent sickness absenteeism or BP episodes. There was moderate-quality evidence that exercise, especially resistance exercise, was the best prevention strategy to reduce the number of people reporting absenteeism due to BP (risk ratio [RR] = 0.10; 95% CI: 0.01 to 0.69). Moderate-quality evidence suggested that resistance and stretching exercises combined with education was the best prevention strategy to reduce pain (RR = 0.80; 95% CI: 0.67 to 0.96) and the number of absenteeism days for BP (standardized mean difference [SMD] = −0.39; 95% CI: −0.77 to −0.02). In conclusion, exercise, especially resistance and stretching exercises, and exercise combined with education were ranked as the best interventions to prevent sickness absenteeism and BP episodes.

## 1. Introduction

Back pain (BP) is a vital public health issue with a high frequency of occurrence. It affects about 80% of people in their lifetime [1] and reoccurs within one year in 70% of people who have recovered from BP [2]. The Global Burden of Disease Study 2017 reported that the number of BP sufferers and years lived with disability increase significantly with age [3].

Because BP is commonly recurrent [2], it might cause frequent pain and work absence (absenteeism). The 12-month prevalence of absenteeism due to BP ranges from 10% [4] to 18% [5]. In addition, employees with chronic back pain are more likely to be absent from work for more than one month of the previous 12 months [6]. Therefore, it is necessary to consider effective strategies to prevent sickness absenteeism and pain among people with BP and determine which prevention strategies are most effective.

According to two previous meta-analyses, which were largely focused on working age populations [7,8], the six most popular prevention strategies for BP are as follows: education, ergonomic adjustments, exercise, back belts, shoe insoles, and multidisciplinary approaches (combinations of these interventions). Exercise alone and exercise combined with education might decrease work absences due to BP [7,8], and these strategies are the best interventions to prevent productivity loss due to BP [8]. However, the types of exercise that might be the best strategies for preventing for absenteeism due to BP are unclear according to two previous meta-analyses [7,8].

Since the latest network meta-analysis was published in 2020 [8], several randomized controlled trials (RCTs) have been published with contradictory results: exercise and exercise with education might not prevent sickness absence due to BP [9,10]. Thus, the goal of this study was to compare the effectiveness of interventions for preventing work absences (sickness absenteeism) due to BP and BP episodes among people with BP. The specific research questions for this study were as follows:

1. Which preventive strategy is most effective in decreasing work absences due to BP (the number of people reporting sickness absenteeism due to BP and the number of days of work absence because of BP) among people with BP?
2. Which preventive strategy is most effective in reducing BP episodes (the number of people reporting BP) among people with BP?

## 2. Materials and Methods

We conducted this study following the PRISMA Extension Statement for Reporting of Systematic Reviews Incorporating Network Meta-analyses of Health Care Interventions (PRISMA-NMA) (Supplementary Materials S11) [11].

The PROSPERO registration number of this study proposal was CRD42022331542.

### 2.1. Study Selection

We included studies if the (1) papers were published in English; (2) papers were RCTs; (3) participants were 18 years old or older without BP or subjects had mild BP but still worked at the baseline; (4) interventions were BP preventive strategies; (5) control group received no intervention (only usual care) or a minimal intervention; and (6) papers reported at least one outcome measure of a work absence due to BP (e.g., the number of people reporting sickness absenteeism due to BP or the number of days of work absence due to BP).

Studies were ineligible if (1) there was no full text; (2) the data were unavailable for extraction and we could not contact the corresponding authors or estimate the data; (3) the study included pregnant women; and (4) the study compared two or more preventive strategies.

### 2.2. Search Strategy

We searched published articles from two previous meta-analyses [7,8], PubMed, Embase and the Cochrane Central Register of Controlled Trials (CENTRAL) with the article type “randomized controlled trials” and the keywords “backache”, “prevention”, “back pain”, “prevent”, and “work absences” since the date last searched in the previous network meta-analysis (24 November 2017) [8]. The last date we searched for papers was in June 2022. We updated our search in January 2023 and October 2023 (Supplementary Materials S1).

### 2.3. Data Collection

Two authors independently searched for papers and examined the titles and abstracts to exclude irrelevant studies and extracted data into a form. A third researcher resolved any disagreements between the two authors through group discussion.

We conducted study selection following the PRISMA 2020 statement [12]. We extracted data on the number of people reporting BP and sickness absenteeism for BP in an intention-to-treat analysis [13] (Supplementary Materials S10). In the case of only a per-protocol analysis or an undefined analysis, we used the available data that were reported.

For missing data, we contacted the corresponding authors or estimated standard deviations [14] if the authors reported only ranges of outcome variables.

### 2.4. Risk of Bias (ROB) and Certainty Assessment

Version 2 of the Cochrane ROB tool for randomized trials [15] was used to evaluate the ROB for each study, and ROB plots were created using the Risk-Of-Bias VISualization (robvis) tool [16]. The certainty assessment for each comparison was conducted based on the Grading of Recommendations Assessment, Development, and Evaluation (GRADE) guidelines [17,18,19]. Two authors assessed the certainty of evidence and ROB independently. Discrepancies between the two authors were resolved by the third author.

### 2.5. Summary Measures

The primary outcome was work absence due to BP, which was defined as the number of people reporting work absences due to BP at follow-up and the number of days of work absence because of BP. The number of people reporting work absences was calculated as a risk ratio (RR) and the associated 95% confidence interval (95%CI). A standardized mean difference (SMD) and 95% CI for continuous data (the number of days of work absence due to BP) was calculated when studies used different questions.

The secondary outcome was BP episodes (pain). This outcome was defined by the number of people reporting BP at follow-up and calculated as an RR and 95%CI.

The *p*-score ranging from 0 to 1 was used to rank interventions in a frequentist network meta-analysis [20]. A higher *p*-score indicated a better intervention [21].

### 2.6. Data Analysis

We used R version 4.3.0 to analyze the data [22]. A frequentist network meta-analysis was performed to compare BP prevention outcomes using the R package Netmeta [23]. We created network graphs for each outcome to visually display the network geometry.

We used both local and global approaches to evaluate inconsistencies in the network of interventions. For the global approach, the Q statistic was calculated to evaluate the inconsistency of the entire network based on the random effects design by-treatment model [24,25]. Between-study variance τ^2^ and the I^2^ statistic were used to measure the heterogeneity of the network across all treatment contrasts [25]. For the local approach, separating indirect from direct evidence (SIDE) was implemented to assess inconsistency [26]. *p*-values of <0.05 suggested statistically significant inconsistencies.

We conducted a sensitivity analysis by excluding studies with a high ROB in the domain of the overall ROB to assess the robustness of the results. Publication bias was detected with the Egger test [25] and comparison-adjusted funnel plots with at least 10 included studies for each outcome variable [27]. A subgroup analysis was performed to define which type(s) of exercise was the best intervention to reduce sickness absenteeism and BP episodes. According to a previous meta-analysis [28] and purpose of the exercises, we classified four types of exercise as follows: (1) resistance or strength exercise to “improve the strength and endurance of skeletal muscles” [29]; (2) aerobic exercise (i.e., walking, cycling) to “improve the efficiency and capacity of the cardiorespiratory system” [29]; (3) stretching exercise to “increase flexibility including passive, static, isometric, ballistic, and proprioceptive neuromuscular facilitation” [30]; and (4) motor control exercise to “improve control and coordination of the spine and pelvis” [31].

## 3. Results

### 3.1. Study Selection

**Figure 1:**
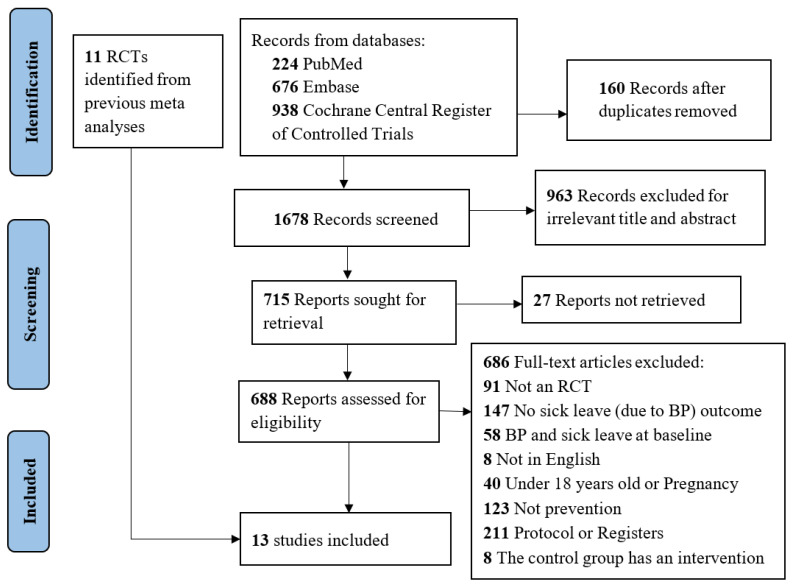
PRISMA flowchart.

The study selection process is presented in Figure 1.

Among 13 included studies in Table 1, 12 studies reported the number of people reporting work absences [9,32,33,34,35,36,37,38,39,40,41,42], 9 studies presented the number of days of work absence [10,32,33,34,36,37,38,39,42], and 10 studies presented the number of people reporting BP at follow-up [9,32,33,35,37,38,39,40,41,42]. Most participants were recruited from community and health facilities and were aged 35~50 years.

### 3.2. Risk of Bias (ROB)

Among 13 RCTs, the overall ROB domains of eight studies were judged to have some concerns [9,10,32,33,35,38,41,42], while those of the 5 other RCTs were determined to have a high ROB [34,36,37,39,40] (Supplementary Materials S2).

### 3.3. Certainty Assessment

Most certainty of evidence assessments for comparisons were low or moderate (Supplementary Materials S7 and S8).

### 3.4. Network Graphs

**Figure 2:**
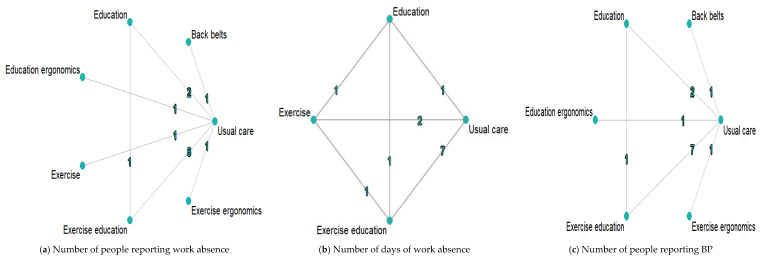
Network graphs of prevention strategies for BP. The number of trials for each comparison is presented on the lines of the network graphs.

Figure 2 presents network graphs of three outcome variables. For the number of people reporting work absences, there were 6 prevention strategies, 18 pairwise comparisons, and 2033 participants. There were 3 prevention strategies, 13 pairwise comparisons and 1421 participants for the number of days of work absence. For the number of people reporting BP, there were 5 prevention strategies, 16 pairwise comparisons, and 1861 participants. Usual care (no prevention for BP) was directly linked to other interventions. 

### 3.5. Treatment Rankings

For the outcome of people reporting work absence, the results of network meta-analysis estimates in Table 2a and Table 3 show that exercise was more likely to decrease the number of people reporting work absence than exercise combined with education (RR = 0.11; 95% CI: 0.02~0.82, *p*-score = 0.98), education (RR = 0.10; 95% CI: 0.01~0.82, *p*-score = 0.47), education combined with ergonomics (RR = 0.10; 95% CI: 0.01~0.80, *p*-score = 0.43), usual care (RR = 0.10; 95%CI: 0.01~0.69, *p*-score = 0.35), and back belts (RR = 0.07; 95%CI: 0.01~0.53, *p*-score = 0.13).

For the outcome of days of work absence, Table 2b and Table 3 show that only exercise combined with education was associated with the number of days of work absence (SMD = −0.39; 95% CI: −0.77~−0.02, *p*-score = 0.77) compared to usual care.

For the outcome of people reporting BP (BP episodes), Table 2c and Table 3 show that exercise combined with education was more likely to reduce the number of people with BP compared to usual care (RR = 0.80; 95% CI: 0.67~0.96, *p*-score = 0.90).

We found no evidence of heterogeneity (τ^2^ = 0; I^2^ = 0%; 95% CI: 0%~64.8%) for the outcome of people reporting work absences. There was evidence of substantial heterogeneity (τ^2^ = 0.21; I^2^ = 82.2%; 95% CI: 66.1%~90.6%) for the outcome of days of work absence and moderate heterogeneity (τ^2^ = 0.03; I^2^ = 56.6%; 95% CI: 4.5%~80.3%) for the outcome of people reporting BP.

There was no total inconsistency in the outcome of people reporting work absences (Q statistics = 2.14; *p* = 0.34), the outcome of days of work absence (Q statistics = 0.77; *p* = 0.68), and the outcome of people reporting BP (Q statistics = 0.54; *p* = 0.77) (global approach) (Supplementary Materials S3). There was no inconsistency indicating disagreement between the indirect and direct evidence within the network (local approach) (*p* > 0.05) (Supplementary Materials S4).

### 3.6. Sensitivity Analysis

To perform a sensitivity analysis, we excluded studies with a high ROB in the overall domain. For the number of people reporting work absences, we could not compare the effectiveness of exercise to back belts, education, education combined with ergonomics, exercise combined with education, and usual care because these interventions were conducted in excluded studies. Exercise combined with education was not associated with the number of days of work absence or the number of people reporting BP, showing that the excluded studies with a high ROB might affect the results of these relationships (Supplementary Materials S5).

### 3.7. Publication Bias

The Egger test and the comparison-adjusted funnel plot revealed no significant publication bias (*p* = 0.56) for the number of people reporting a work absence outcome. For the number of people who had a BP outcome, significant publication bias might present as funnel asymmetry and Egger test (*p* = 0.002) (Supplementary Materials S6).

### 3.8. Subgroup Analysis

For comparisons of all types of exercise interventions with usual care, resistance exercise was associated with the number of people reporting work absences (RR = 0.10; 95% CI: 0.01~0.69; *p*-score = 0.97), followed by resistance and stretching exercises combined with education (RR = 0.74; 95% CI: 0.55~0.99; *p*-score = 0.64). Compared to usual care, only a combination of resistance, stretching exercises, and education was correlated with the days of work absence (SMD = −0.47; 95% CI: −0.87~−0.07; *p*-score = 0.78) and the number of people reporting BP (RR = 0.67; 95% CI: 0.54~0.82; *p*-score = 0.88) (Supplementary Materials S9).

## 4. Discussion

Exercise was ranked the best prevention strategy to reduce the number of people reporting work absences due to BP (RR = 0.10; 95% CI: 0.01~0.69). Exercise combined with education was ranked the best prevention strategy to reduce the number of days of work absence due to BP (SMD = −0.39; 95% CI: −0.77~−0.02) and the number of people reporting BP (RR = 0.80; 95% CI: 0.67~0.96). Compared to usual care, resistance exercise was associated with the number of people reporting work absences due to BP (RR = 0.10; 95% CI: 0.01 ~ 0.69). Only a combination of resistance, stretching exercises, and education was correlated with the days of work absence due to BP (SMD = −0.47; 95% CI: −0.87~−0.07) and the number of people reporting BP (RR = 0.67; 95%CI: 0.54~0.82).

Consistent with two previous meta-analyses [7,8], this study found that only exercise was associated with the number of people reporting work absences. This finding was contrary to another meta-analysis [43]. This difference could be attributed to the eligibility criteria of that meta-analysis. To precisely evaluate the effectiveness of interventions to decrease work absences, we had stricter inclusion criteria because we excluded studies with a non-RCT design or children as study participants.

Exercise combined with education, exercise combined with ergonomics, education combined with ergonomics, education, and back belts were not likely to reduce the number of people reporting work absences. These results were consistent with those of previous meta-analyses [7,8]. Exercise was significantly effective compared to exercise combined with education, education combined with ergonomics, education, and back belts in reducing the number of people reporting work absences due to BP. These results are consistent with the previous network meta-analysis [8].

In contrast to previous meta-analyses [44,45], our study demonstrated that exercise was ineffective in decreasing the number of days of work absence caused by BP compared to usual care. This relationship may be explained by the previous meta-analysis [45] pooling studies reporting sick days caused by all diseases in patients with BP. Another possible explanation is that we included 10 studies with a long follow-up period (of 6~36 months), while the previous meta-analysis [44] pooled only two studies with medium-term follow-up (of 6~24 months). These findings might support the critical issue raised in the previous meta-analysis [7] that the effect of exercise on BP prevention might decrease over time, and ongoing exercise should be maintained to reduce work absences.

Exercise combined with education was significantly more effective than usual care in reducing the number of days of work absence. This result might conflict with the association between exercise and the number of days of work absence in this study. The reason might be that exercise combined with education might change attitudes or behaviors of participants about BP and encourage them to return to work sooner.

Like previous meta-analyses [7,8], our study updated the effectiveness of all BP prevention strategies on the number of people reporting work absences. Compared to previous meta-analyses [7,8], our new findings were aimed to identify the most effective among all prevention strategies for BP in terms of the number of days of work absence outcome and the specific types of exercise that contributed most to preventing work absences and BP episodes.

Our study had several limitations. First, most comparisons had low- or moderate-quality evidence, and several included studies had high risks of bias in the overall domain in this study. Therefore, it might not have accurately estimated the true effect of the interventions, especially exercise, on work absences. Second, several RCTs did not define whether the interventions were prevention or treatment [7], so we could not include those studies. Third, our study included trials with different numbers of follow-up months and limited types of exercise. Thus, it is uncertain whether the long-term effects of all types of exercise persist to prevent work absences. Fourth, a small number of trials and limited types of interventions for BP were included because our primary outcome was work absences.

## 5. Conclusions

In conclusion, exercise, especially resistance and stretching exercises, and exercise combined with education were ranked the best strategies for preventing work absences (sickness absenteeism) due to BP and BP episodes. Further studies on the frequency and intensity of all types of exercise are required to define which kinds of exercise are effective interventions for preventing work absences caused by BP in long-term follow-up (over one year).

## Figures and Tables

**Table 1:** Characteristics of the included studies.

No	Author, Year	Participants (Mean Age; SD or Range); Sex	Outcome	Intervention and Control Groups	Follow-Up Months
1	Lønn 1999 [32]	81 participants recruited from referrals and advertisement; (39.4 years; 19.2–49.8); 46% male	Number of subjects reporting sick leave Number of days of sick leave due to BP Number of episodes of low BP	Exercise and education: resistance (strength training of muscles), stretching exercises Control group: no intervention	12 months
2	Glomsrød 2001 [33]	81 participants recruited from referrals and advertisement; (39.4 years; 19.2–49.8); 46% male	Number of subjects reporting sick leave Number of days of sick leave due to episodes of BP Number of episodes of low BP	Exercise and education: resistance (strength training of muscles), stretching exercises Control group: no intervention	36 months
3	Gundewall 1993 [34]	69 nurses and nurse’s aides; (37.5 years; 10.5); 1% male	Number of subjects with work absence Number of lost work-days due to BP	Exercise: resistance exercises (back muscle exercises to increase endurance and muscle strength) Control: no intervention	13 months
4	Ijzelenberg 2007 [35]	489 workers from physically demanding jobs; (41.3 years; 9.7); 98% male	Number of people absent from work during the previous six months due to BP Number of participants with low BP	Education and ergonomic adjustments Control: usual care	12 months
5	Kellett 1991 [36]	111 employees of kitchen unit production; (41.7 years; 10.1); 70% male	Number of people with sick leave because of BP Number of sick leave days due to BP	Exercise and education: resistance, stretching exercises Control: no intervention	18 months
6	Soukup 1999 [37]	77 outpatients from general clinical practices; (39.6 years; 21.2–49.8); 47% male	Number of participants with sick leave for BP Number of days of sick leave due to BP Number of participants with recurrence of low BP episodes	Exercise and education: (Mensendieck) resistance, stretching exercises Control: no intervention	12 months
7	Soukup 2001 [38]	77 outpatients from general clinical practices; (37.7 years; 8.0); 47% male	Number of participants with sick leave due to BP Number of days of sick leave due to BP Number of subjects with recurrent episodes of low BP	Exercise and education: Mensendieck resistance, stretching exercises Control: no intervention	36 months
8	van Poppel 1998 [39]	312 airline employees; (35.1 years; 7.8); Not available	Number of subjects with sick leave because of BP Number of days per month of sick leave because of BP Number of subjects with low BP	Education and back belts Education Back belts Control: no intervention	6 months
9	Warming 2008 [40]	181 hospital nurses; (34.8 years; 9.3); Not available	Number of subjects with sick leave because of BP Number of subjects who experienced low BP	Education Exercise: aerobic and resistance exercises Control: no intervention	12 months
10	Roussel 2015 [41]	69 hospital workers; (40.8 years; not available); 18% male	Number of workers with work absenteeism because of BP Number of subjects with low BP	Exercise and ergonomics: (stabilization) motor control Control: no intervention	6 months
11	Chaléat-Valayer 2016 [42]	342 healthcare workers; (47.2 years; no information); 23% male	Percentage of participants with sick leave related to chronic low BP (pain for >3 months) Duration of sick leave due to BP episodes (days) Percentage of participants with ≥1 recurrence of low BP with sick leave	Exercise and education: stretching exercises Control: no intervention	24 months
12	Suni 2018 [10]	219 female healthcare workers; (46.4 years; 6.8); 0% male	Number of days absent from work due to low BP	Exercise: resistance, stretching exercises Exercise and counselling *: resistance, stretching exercises Counselling *: education Control: no intervention	12 months
13	Ferreira 2021 [9]	111 participants recruited from primary care and community facilities; (50.2 years; 13.1); 50% male	Number of people with sick leave because of BP Number of participants with recurrence of low BP	Exercise and education: resistance exercise Control: minimal intervention	12 months

* Counselling was considered a type of education.

**Table 2:** League tables of pairwise meta-analysis estimates (the upper triangle) and network meta-analysis estimates (the lower triangle). (**a**) Number of people reporting work absence. (**b**) Number of days of work absence. (**c**) Number of people reporting BP.

(**a**)						
Exercise					*0.10 (0.01;0.69)*	
*0.11 (0.02; 0.82)*	Exercise education		0.38 (0.08;1.81)		0.86 (0.68; 1.08)	
0.14 (0.01; 1.94)	1.28 (0.23; 7.10)	Exercise ergonomics			0.67 (0.12; 3.65)	
*0.10 (0.01; 0.82)*	0.93 (0.50; 1.74)	0.73 (0.12; 4.42)	Education		0.86 (0.47; 1.57)	
*0.10 (0.01; 0.80)*	0.90 (0.47; 1.74)	0.70 (0.12; 4.31)	0.96 (0.41; 2.26)	Education ergonomics	0.95 (0.51; 1.76)	
*0.10 (0.01; 0.69)*	0.85 (0.68; 1.07)	0.67 (0.12; 3.65)	0.91 (0.51; 1.64)	0.95 (0.51; 1.76)	Usual care	0.69 (0.35; 1.37)
*0.07 (0.01; 0.53)*	0.59 (0.29; 1.21)	0.46 (0.07; 2.89)	0.63 (0.26; 1.55)	0.65 (0.26; 1.65)	0.69 (0.35; 1.37)	Back belts
(**b**)						
Exercise education		−0.26 (−1.28; 0.75)		−0.14 (−1.17; 0.89)	−0.37 (−0.75; 0.01)	
−0.16 (−0.88; 0.57)		Exercise		0.12 (−0.91; 1.15)	−0.21 (−0.94; 0.51)	
−0.19 (−1.09; 0.71)		−0.03 (−1.00; 0.93)		Education	0.00 (−1.03; 1.03)	
*−0.39 (−0.77; −0.02)*		−0.24 (−0.92; 0.44)		−0.20 (−1.09; 0.69)	Usual care	
(**c**)						
Exercise education	*0.81 (0.67;0.97)*				0.60 (0.34; 1.07)	
*0.80 (0.67; 0.96)*	Usual care	0.96 (0.61; 1.52)		0.93 (0.63; 1.38)	0.92 (0.66; 1.28)	0.50 (0.05; 5.29)
0.77 (0.47; 1.26)	0.96 (0.61; 1.52)	Back belts				
0.75 (0.48; 1.15)	0.93 (0.63; 1.38)	0.97 (0.53; 1.78)		Education ergonomics		
0.72 (0.51; 1.02)	0.90 (0.65; 1.23)	0.93 (0.53; 1.63)		0.96 (0.58; 1.60)	Education	
0.40 (0.04; 4.27)	0.50 (0.05; 5.29)	0.52 (0.05; 5.75)		0.54 (0.05; 5.88)	0.56 (0.05; 6.03)	Exercise ergonomics

RRs < 1 indicate that the column intervention is more effective than the row intervention. SMDs < 0 indicate that the column intervention is more effective than the row intervention. Significant effects between two interventions are presented in italic font.

**Table 3:** Prevention strategies for BP ranked according to work absences and the number of people reporting BP.

No.	Intervention	Number of People Reporting Work Absence		Number of Days of Work Absence		Number of People Reporting BP	
		*p*-Score	Rank	*p*-Score	Rank	*p*-Score	Rank
1	Exercise	0.98	1	0.54	2	-	-
2	Exercise education	0.58	2	0.77	1	0.90	1
3	Exercise ergonomics	0.57	3	-	-	0.28	6
4	Education	0.47	4	0.49	3	0.36	5
5	Education ergonomics	0.43	5	-	-	0.43	4
6	Usual care	0.35	6	0.20	4	0.54	2
7	Back belts	0.13	7	-	-	0.49	3

Higher *p*-scores indicate better interventions and higher ranks.

## Data Availability

The data of this study are available in Supplementary Materials.

## Supplementary Materials

The following supporting information can be downloaded at: https://www.mdpi.com/2254-9625/13/12/200/s1. Supplementary Materials S1: Search Strategy, Supplementary Materials S2: Risk of bias summary, Supplementary Materials S3: Global Inconsistency of All Outcomes, Supplementary Materials S4: Local inconsistency within the network, Supplementary Materials S5: Sensitivity Analysis, Supplementary Materials S6: Comparison-Adjusted Funnel Plot, Supplementary Materials S7: Certainty of Direct Evidence Assessment, Supplementary Materials S8: Certainty of Network Evidence Assessment, Supplementary Materials S9: Subgroup analysis, Supplementary Materials S10: Summary of data, Supplementary Materials S11: PRISMA NMA Checklist of Items to Include When Reporting A Systematic Review Involving a Network Meta-analysis.
