# Supplementary material for: Prevention of Work Absence Due to Back Pain: A Network Meta-Analysis
Source: Eur J Investig Health Psychol Educ. 2023 Dec 10;13(12):2891–903. doi: 10.3390/ejihpe13120200 (PMC10871117; doi:10.3390/ejihpe13120200)
Supplement: Supplementary file 1 [file ejihpe-13-00200-s001.zip › ejihpe-2710519-supplementary.pdf]

Prevention of Work Absence Due to Back Pain: A Network Meta-Analysis

Supplementary Materials S1: Search Strategy 1
Supplementary Materials S2: Risk of bias summary 6
Supplementary Materials S3: Global Inconsistency of All Outcomes 8
Supplementary Materials S4: Local inconsistency within the network (Netsplit Analysis of Inconsistency) 9
Supplementary Materials S5: Sensitivity Analysis 13
Supplementary Materials S6: Comparison-Adjusted Funnel Plot 15
Supplementary Materials S7: Certainty of Direct Evidence Assessment 16
Supplementary Materials S8: Certainty of Network Evidence Assessment 18
Supplementary Materials S9: Subgroup analysis 23
Supplementary Materials S10: Summary of data 26
Supplementary Materials S11: PRISMA NMA Checklist of Items to Include When Reporting A Systematic Review Involving a Network Meta-analysis 29

Supplementary Materials S1: Search Strategy

Keywords for randomized controlled trials since the date last searched of the previous network meta-analysis (24 November 2017)

1.1 PubMed

1) backache + prevention + RCT: 11 results

(backache + prevention[Title/Abstract]) AND (("2017"[Date - Publication] : "3000"[Date - Publication]))

2) back pain + prevent +RCT: 17 results

(back pain+prevent[Title/Abstract]) AND (("2017"[Date - Publication] : "3000"[Date - Publication]))

3) back pain + prevention + RCT: 21 results

(back pain+prevention[Title/Abstract]) AND (("2017"[Date - Publication] : "3000"[Date - Publication]))

4) back AND pain AND prevent + RCT: 43 results

(back[Title/Abstract] AND pain[Title/Abstract] AND prevent[Title/Abstract]) AND (("2017"[Date - Publication] : "3000"[Date - Publication]))

5) backache + prevention + sick leave + RCT: 5 results

(backache + prevention + sick leave[Title/Abstract]) AND (("2017"[Date - Publication] : "3000"[Date - Publication]))

6) "low back pain"+"prevent"+"sick leave": 12 results

(("low back pain"[All Fields] AND "prevent"[All Fields]) AND "sick leave"[All Fields]) AND (randomizedcontrolledtrial[Filter])

7) backache + prevent + RCT: 49 results

(backache + prevent[Title/Abstract]) AND (("2017/11/24"[Date - Publication] : "3000"[Date - Publication]))

8) backache + sick leave + RCT: 30 results

(backache + sick leave[Title/Abstract]) AND (("2017/11/24"[Date - Publication] : "3000"[Date - Publication]))

9) backache + absenteeism + RCT: 9 results

(backache + absenteeism[Title/Abstract]) AND (("2017/11/24"[Date - Publication] : "3000"[Date - Publication]))

10) back pain + absenteeism + RCT: 9 results

(back pain + absenteeism[Title/Abstract]) AND (("2017/11/24"[Date - Publication] : "3000"[Date - Publication]))

11) backache + work absence + RCT: 9 results

(backache + work absence[Title/Abstract]) AND (("2017/11/24"[Date - Publication] : "3000"[Date - Publication]))

12) back pain + work absence + RCT: 9 results

(back pain + work absence[Title/Abstract]) AND (("2017/11/24"[Date - Publication] : "3000"[Date - Publication]))

1.2 Embase

1) backache + prevention + RCT: 297 results

('backache'/exp OR backache) AND ('prevention'/exp OR prevention) AND ([controlled clinical trial]/lim OR [randomized controlled trial]/lim) AND ([article]/lim OR [article in press]/lim OR [data papers]/lim) AND [english]/lim AND [adult]/lim AND [2017-2022]/py

('backache'/exp OR backache) AND + AND ('prevention'/exp OR prevention) AND [randomized controlled trial]/lim AND [english]/lim AND ([adult]/lim OR [young adult]/lim OR [middle aged]/lim OR [aged]/lim OR [very elderly]/lim) AND [humans]/lim AND [embase]/lim AND [06-06-2022]/sd NOT [25-01-2023]/sd

2) low back pain + prevent + sick leave: 0 results

'low back pain+prevent+sick leave' OR (low AND ('back'/exp OR back) AND pain+prevent+sick AND leave)

low AND back AND pain AND prevent AND + AND sick AND leave AND [randomized controlled trial]/lim AND [english]/lim AND ([adult]/lim OR [aged]/lim) AND [humans]/lim AND [embase]/lim AND [06-06-2022]/sd NOT [25-01-2023]/sd

3) back pain + prevent + RCT: 10 results

('back pain+prevent' OR (('back'/exp OR back) AND pain+prevent)) AND ([controlled clinical trial]/lim OR [randomized controlled trial]/lim)

back AND pain AND + AND prevent AND [randomized controlled trial]/lim AND [english]/lim AND ([adult]/lim OR [aged]/lim) AND [humans]/lim AND [embase]/lim AND [06-06-2022]/sd NOT [25-01-2023]/sd

4) back pain + prevention + RCT: 32 results

('back pain+prevention' OR (('back'/exp OR back) AND pain+prevention)) AND ([controlled clinical trial]/lim OR [randomized controlled trial]/lim)

back AND pain AND + AND prevention AND [randomized controlled trial]/lim AND [english]/lim AND ([adult]/lim OR [aged]/lim) AND [humans]/lim AND [embase]/lim AND [06-06-2022]/sd NOT [25-01-2023]/sd

5) back AND pain AND prevent + RCT (in English): 211 results

('back'/exp OR back) AND ('pain'/exp OR pain) AND prevent AND ([adult]/lim OR [young adult]/lim OR [middle aged]/lim OR [aged]/lim OR [very elderly]/lim) AND ([adult]/lim OR [aged]/lim) AND ([controlled clinical trial]/lim OR [randomized controlled trial]/lim) AND ([article]/lim OR [article in press]/lim OR [data papers]/lim) AND [english]/lim AND [adult]/lim AND [humans]/lim

back AND pain AND prevent AND [randomized controlled trial]/lim AND [english]/lim AND ([adult]/lim OR [aged]/lim) AND [humans]/lim AND [embase]/lim AND [06-06-2022]/sd NOT [25-01-2023]/sd

6) backache + prevention + sick leave + RCT: 47 results

('backache'/exp OR backache) AND ('prevention'/exp OR prevention) AND ('sick leave'/exp OR 'sick leave' OR (sick AND leave)) AND ([controlled clinical trial]/lim OR [randomized controlled trial]/lim) AND ([article]/lim OR [article in press]/lim OR [data papers]/lim) AND [english]/lim AND [adult]/lim

back AND pain AND prevent AND ([adult]/lim OR [aged]/lim) AND [embase]/lim AND [randomized controlled trial]/lim AND [english]/lim AND [adult]/lim AND [humans]/lim AND [06-06-2022]/sd NOT [25-01-2023]/sd

7) backache + prevent + RCT: 8 results

backache AND + AND prevent AND [randomized controlled trial]/lim AND [english]/lim AND [adult]/lim AND [humans]/lim AND [embase]/lim AND [06-06-2022]/sd NOT [25-01-2023]/sd

8) backache + sick leave + RCT: 5 results

backache AND + AND sick AND leave AND [randomized controlled trial]/lim AND [english]/lim AND [adult]/lim AND [humans]/lim AND [embase]/lim AND [24-11-2017]/sd NOT [25-01-2023]/sd

9) backache + absenteeism + RCT: 10 results

backache AND + AND absenteeism AND [randomized controlled trial]/lim AND [english]/lim AND ([adult]/lim OR [aged]/lim) AND [humans]/lim AND [embase]/lim AND [24-11-2017]/sd NOT [25-01-2023]/sd

10) back pain + absenteeism + RCT: 29 results

('back'/exp OR back) AND ('pain'/exp OR pain) AND ('absenteeism'/exp OR absenteeism) AND [randomized controlled trial]/lim AND [english]/lim AND ([adult]/lim OR [aged]/lim) AND [humans]/lim AND [embase]/lim AND [24-11-2017]/sd NOT [25-01-2023]/sd

11) backache + work absence + RCT: 14 results

('backache'/exp OR backache) AND + AND ('work'/exp OR work) AND ('absence'/exp OR absence) AND [randomized controlled trial]/lim AND [english]/lim AND [adult]/lim AND [humans]/lim AND [embase]/lim AND [24-11-2017]/sd NOT [27-01-2023]/sd

12) back pain + work absence + RCT: 13 results

back AND pain AND + AND work AND absence AND [randomized controlled trial]/lim AND [english]/lim AND [adult]/lim AND [humans]/lim AND [embase]/lim AND [24-11-2017]/sd NOT [27-01-2023]/sd

1.3 Cochrane Library: Cochrane Central Register of Controlled Trials (CENTRAL)

1) backache + prevention + RCT: 147 results

(backache) AND (prevention) (Word variations have been searched) with Publication Year from 2017 to 2022, with Cochrane Library publication date Between Nov 2017 and Jun 2022, in Trials

backache AND prevention in Title Abstract Keyword - with Publication Year from 2022 to 2023, with Cochrane Library publication date Between Jun 2022 and Jan 2023, in Trials (Word variations have been searched)

2) back pain + prevention + RCT: 506 results

("back pain") AND (prevention) with Publication Year from 2017 to 2022, with Cochrane Library publication date Between Nov 2017 and Jun 2022, in Trials (Word variations have been searched)

back pain AND prevention in Title Abstract Keyword - with Publication Year from 2022 to 2023, with Cochrane Library publication date Between Jun 2022 and Jan 2023, in Trials (Word variations have been searched)

3) backache + sick leave + RCT: 5 results

backache AND sick leave in Title Abstract Keyword - with Publication Year from 2017 to 2023, with Cochrane Library publication date Between Nov 2017 and Jan 2023, in Trials (Word variations have been searched)

4) backache + absenteeism + RCT: 9 results

backache AND absenteeism in Title Abstract Keyword - with Publication Year from 2017 to 2023, with Cochrane Library publication date Between Nov 2017 and Jan 2023, in Trials (Word variations have been searched)

5) backache + work absence + RCT: 9 results

backache AND work absence in Title Abstract Keyword - with Publication Year from 2017 to 2023, with Cochrane Library publication date Between Nov 2017 and Jan 2023, in Trials (Word variations have been searched)

6) back pain + sick leave + RCT: 102 results

back pain AND sick leave in Title Abstract Keyword - with Publication Year from 2017 to 2023, with Cochrane Library publication date Between Nov 2017 and Jan 2023, in Trials (Word variations have been searched)

7) back pain + absenteeism + RCT: 71 results

back pain AND absenteeism in Title Abstract Keyword - with Publication Year from 2017 to 2023, with Cochrane Library publication date Between Nov 2017 and Jan 2023, in Trials (Word variations have been searched)

8) back pain + work absence + RCT: 89 results

back pain AND work absence in Title Abstract Keyword - with Publication Year from 2017 to 2023, with Cochrane Library publication date Between Nov 2017 and Jan 2023, in Trials (Word variations have been searched)

Supplementary Materials S2: Risk of bias summary

2.1 Individually randomized controlled trials

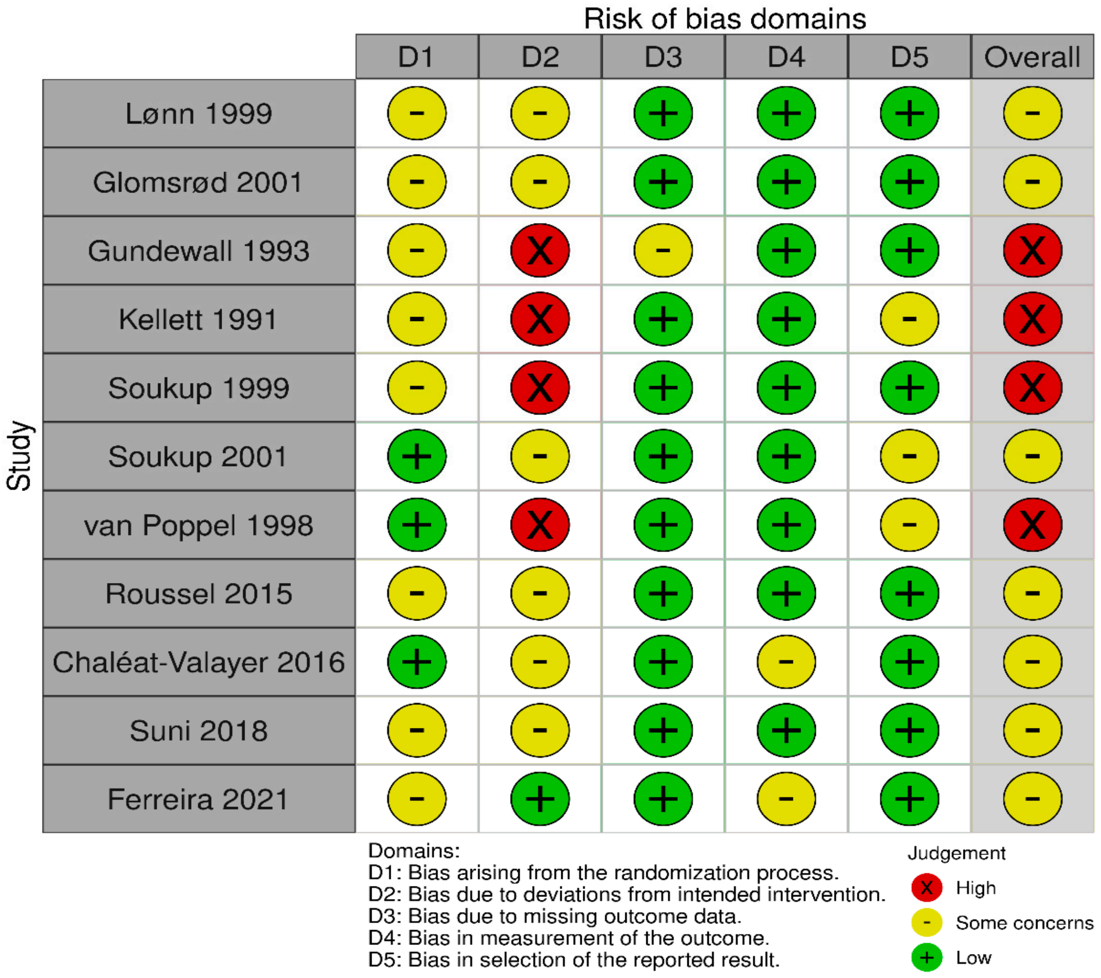

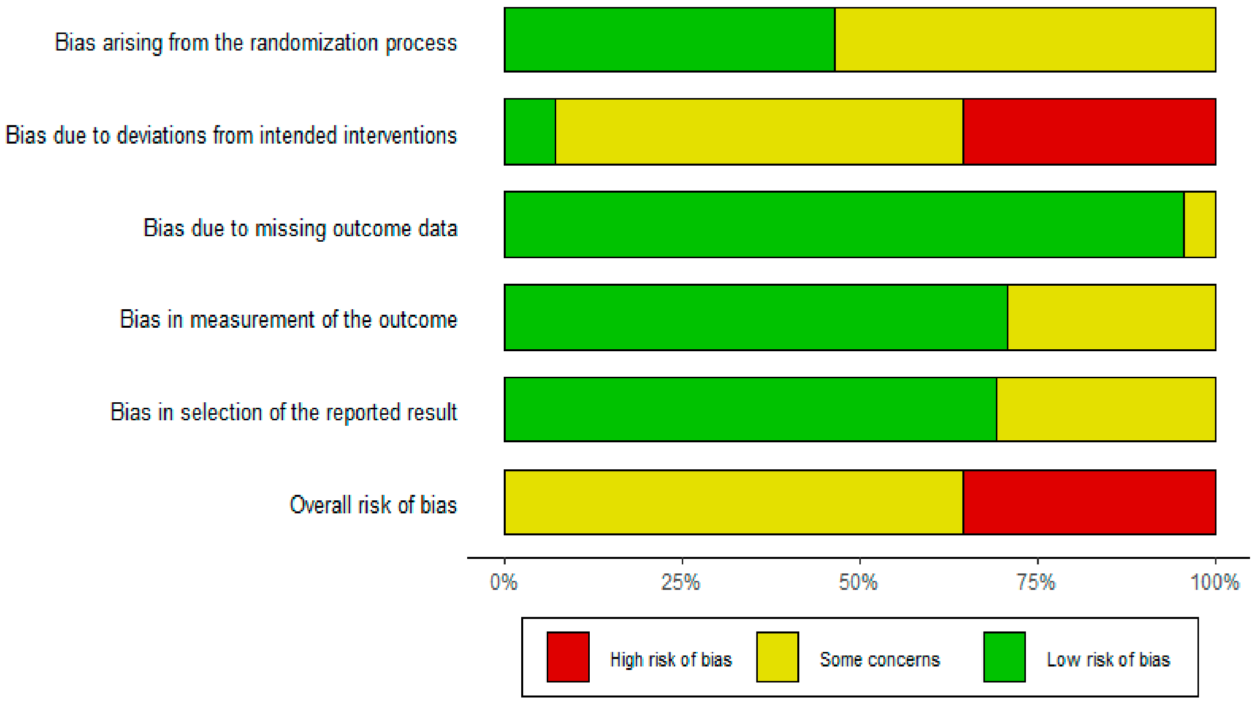

2.2 Cluster randomized controlled trials

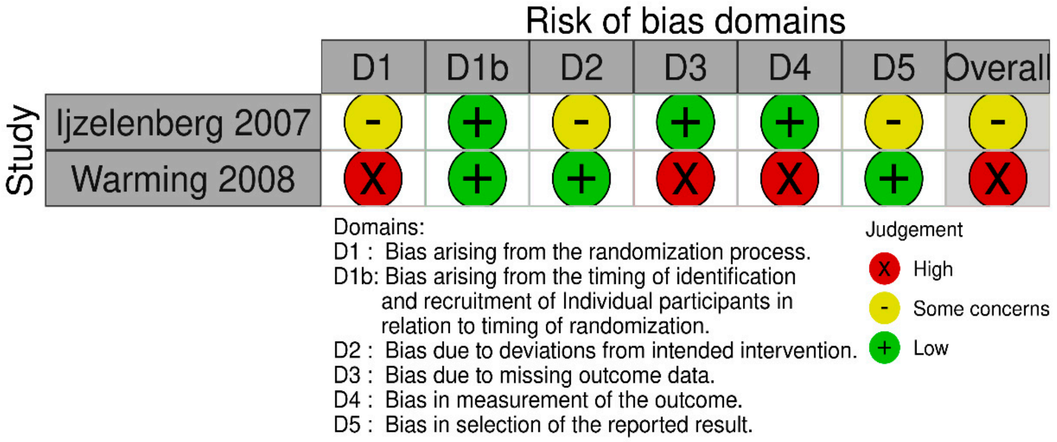

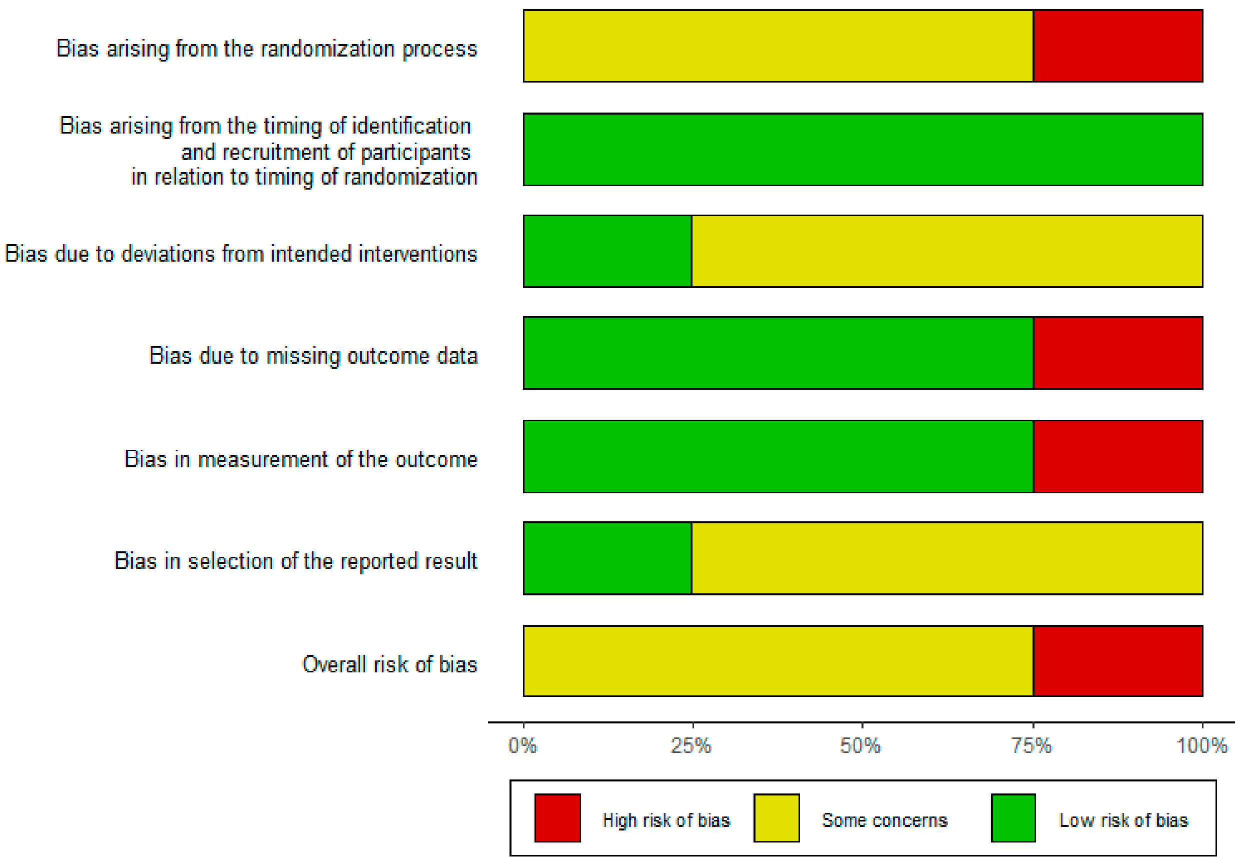

Supplementary Materials S3: Global Inconsistency of All Outcomes

Outcome	Q statistics	P-value	tau.within	tau2.within
Number of people reporting work absence	2.14 (df = 2)	0.3427	0	0
Number of days of sick leave	0.77 (df = 2)	0.6803	0.5113	0.2614
Number of people who had BP	0.54 (df = 2)	0.7650	0.1852	0.0343

Supplementary Materials S4: Local inconsistency within the network (Netsplit Analysis of Inconsistency)

4.1 Number of people reporting a work absence due to BP

comparison	- Treatment comparison
k	- Number of studies providing direct evidence
prop	- Direct evidence proportion
nma	- Estimated treatment effect (RR) in network meta-analysis
direct	- Estimated treatment effect (RR) derived from direct evidence
indir.	- Estimated treatment effect (RR) derived from indirect evidence
RoR	- Ratio of Ratios (direct versus indirect)
z	- z-value of test for disagreement (direct versus indirect)
p-value	- p-value of test for disagreement (direct versus indirect)

Random effects model:

comparison	*k*	*prop*	*nma*	*95%-CI*	*direct*	*95%-CI*	*indir.*	*95%-CI*	*RoR*	*95%-CI*	*z*	*p-value*
Back belts:Education	0	0	1.58	[0.64; 3.90]	.	.	1.58	[0.64; 3.90]	.	.	.	.
Back belts:Education ergonomics	0	0	1.53	[0.61; 3.84]	.	.	1.53	[0.61; 3.84]	.	.	.	.
Back belts:Exercise	0	0	15.17	[1.87; 122.70]	.	.	15.17	[1.87; 122.70]	.	.	.	.
Back belts:Exercise education	0	0	1.70	[0.83; 3.48]	.	.	1.70	[0.83; 3.48]	.	.	.	.
Back belts:Exercise ergonomics	0	0	2.17	[0.35; 13.55]	.	.	2.17	[0.35; 13.55]	.	.	.	.
Back belts:Usual care	1	1.00	1.44	[0.73; 2.86]	1.44	[0.73; 2.86]	.	.	.	.	.	.
Education:Education ergonomics	0	0	0.96	[0.41; 2.26]	.	.	0.96	[0.41; 2.26]	.	.	.	.
Education:Exercise	0	0	9.57	[1.22; 75.20]	.	.	9.57	[1.22; 75.20]	.	.	.	.
Education:Exercise education	1	0.16	1.07	[0.57; 2.00]	2.65	[0.55; 12.74]	0.90	[0.46; 1.78]	2.93	[0.53; 16.22]	1.23	0.2172
Education:Exercise ergonomics	0	0	1.37	[0.23; 8.27]	.	.	1.37	[0.23; 8.27]	.	.	.	.
Education:Usual care	2	0.96	0.91	[0.51; 1.64]	0.86	[0.47; 1.57]	3.33	[0.19; 59.12]	0.26	[0.01; 4.90]	-0.90	0.3679
Education ergonomics:Exercise	0	0	9.93	[1.25; 78.78]	.	.	9.93	[1.25; 78.78]	.	.	.	.
Education ergonomics:Exercise education	0	0	1.11	[0.57; 2.15]	.	.	1.11	[0.57; 2.15]	.	.	.	.
Education ergonomics:Exercise ergonomics	0	0	1.42	[0.23; 8.67]	.	.	1.42	[0.23; 8.67]	.	.	.	.
Education ergonomics:Usual care	1	1.00	0.95	[0.51; 1.76]	0.95	[0.51; 1.76]	.	.	.	.	.	.
Exercise:Exercise education	0	0	0.11	[0.02; 0.82]	.	.	0.11	[0.02; 0.82]	.	.	.	.
Exercise:Exercise ergonomics	0	0	0.14	[0.01; 1.94]	.	.	0.14	[0.01; 1.94]	.	.	.	.
Exercise:Usual care	1	1.00	0.10	[0.01; 0.69]	0.10	[0.01; 0.69]	.	.	.	.	.	.
Exercise education:Exercise ergonomics	0	0	1.28	[0.23; 7.10]	.	.	1.28	[0.23; 7.10]	.	.	.	.

comparison	*k*	*prop*	*nma*	*95%-CI*	*direct*	*95%-CI*	*indir.*	*95%-CI*	*RoR*	*95%-CI*	*z*	*p-value*
Exercise education:Usual care	8	1.00	0.85	[0.68; 1.07]	0.86	[0.68; 1.08]	0.03	[0.00; 3.36]	26.30	[0.25; 2721.62]	1.38	0.1672
Exercise ergonomics:Usual care	1	1.00	0.67	[0.12; 3.65]	0.67	[0.12; 3.65]	.	.	.	.	.	.

4.2 Number of days of sick leave caused by BP

comparison - Treatment comparison

k - Number of studies providing direct evidence

prop - Direct evidence proportion

nma - Estimated treatment effect (SMD) in network meta-analysis

direct - Estimated treatment effect (SMD) derived from direct evidence

indir. - Estimated treatment effect (SMD) derived from indirect evidence

Diff - Difference between direct and indirect treatment estimates

z - z-value of test for disagreement (direct versus indirect)

p-value - p-value of test for disagreement (direct versus indirect)

Random effects model:

comparison	*k*	*prop*	*nma*	*direct*	*indir.*	*Diff*	*z*	*p-value*
Education:Exercise	1	0.87	0.03	-0.12	1.06	-1.19	-0.82	0.4117
Education:Exercise education	1	0.76	0.19	0.14	0.35	-0.21	-0.20	0.8450
Education:Usual care	1	0.75	-0.20	0.00	-0.82	0.82	0.78	0.4343
Exercise:Exercise education	1	0.50	0.16	0.26	0.05	0.22	0.29	0.7692
Exercise:Usual care	2	0.88	-0.24	-0.21	-0.42	0.21	0.20	0.8447
Exercise education:Usual care	7	0.98	-0.39	-0.37	-1.54	1.17	0.82	0.4115

4.3 Number of people who had BP

comparison - Treatment comparison
k - Number of studies providing direct evidence
prop - Direct evidence proportion
nma - Estimated treatment effect (RR) in network meta-analysis
direct - Estimated treatment effect (RR) derived from direct evidence
indir. - Estimated treatment effect (RR) derived from indirect evidence
RoR - Ratio of Ratios (direct versus indirect)
z - z-value of test for disagreement (direct versus indirect)
p-value - p-value of test for disagreement (direct versus indirect)

Random effects model:

comparison	*k*	*prop*	*nma*	*95%-CI*	*direct*	*95%-CI*	*indir.*	*95%-CI*	*RoR*	*95%-CI*	*z*	*p-value*
Back belts:Education	0	0	0.93	[0.53; 1.63]	.	.	0.93	[0.53; 1.63]	.	.	.	.
Back belts:Education ergonomics	0	0	0.97	[0.53; 1.78]	.	.	0.97	[0.53; 1.78]	.	.	.	.
Back belts:Exercise education	0	0	1.30	[0.79; 2.13]	.	.	1.30	[0.79; 2.13]	.	.	.	.
Back belts:Exercise ergonomics	0	0	0.52	[0.05; 5.75]	.	.	0.52	[0.05; 5.75]	.	.	.	.
Back belts:Usual care	1	1.00	1.04	[0.66; 1.65]	1.04	[0.66; 1.65]	.	.	.	.	.	.
Education:Education ergonomics	0	0	1.04	[0.63; 1.73]	.	.	1.04	[0.63; 1.73]	.	.	.	.
Education:Exercise education	1	0.36	1.39	[0.98; 1.97]	1.67	[0.94; 2.97]	1.26	[0.81; 1.95]	1.33	[0.64; 2.73]	0.76	0.4462
Education:Exercise ergonomics	0	0	0.56	[0.05; 6.03]	.	.	0.56	[0.05; 6.03]	.	.	.	.
Education:Usual care	2	0.92	1.12	[0.81; 1.53]	1.08	[0.78; 1.51]	1.59	[0.52; 4.88]	0.68	[0.21; 2.20]	-0.64	0.5230
Education ergonomics:Exercise education	0	0	1.34	[0.87; 2.07]	.	.	1.34	[0.87; 2.07]	.	.	.	.
Education ergonomics:Exercise ergonomics	0	0	0.54	[0.05; 5.88]	.	.	0.54	[0.05; 5.88]	.	.	.	.
Education ergonomics:Usual care	1	1.00	1.07	[0.72; 1.60]	1.07	[0.72; 1.60]	.	.	.	.	.	.
Exercise education:Exercise ergonomics	0	0	0.40	[0.04; 4.27]	.	.	0.40	[0.04; 4.27]	.	.	.	.

comparison	*k*	*prop*	*nma*	*95%-CI*	*direct*	*95%-CI*	*indir.*	*95%-CI*	*RoR*	*95%-CI*	*z*	*p-value*
Exercise education:Usual care	7	0.99	0.80	[0.67; 0.96]	0.81	[0.67; 0.97]	0.45	[0.07; 2.90]	1.80	[0.28; 11.76]	0.61	0.5390
Exercise ergonomics:Usual care	1	1.00	2.00	[0.19; 21.16]	2.00	[0.19; 21.16]	.	.	.	.	.	.

Supplementary Materials S5: Sensitivity Analysis

5.1 Number of people reporting a work absence due to BP:

Studies of high risk of bias of overall domains (Gundewall 1993, Kellett 1991, Soukup 1999, van Poppel 1998, Warming 2008) were excluded.

Comparison	All studies RR (95% CI)	Excluding studies RR (95% CI)
Back belts:Education	1.58 (0.64; 3.90)	No studies
Back belts:Education ergonomics	1.53 (0.61; 3.84)	No studies
Back belts:Exercise	**15.17 (1.87; 122.70)**	No studies
Back belts:Exercise education	1.70 (0.83; 3.48)	No studies
Back belts:Exercise ergonomics	2.17 (0.35; 13.55)	No studies
Back belts:Usual care	1.44 (0.73; 2.86)	No studies
Education:Education ergonomics	0.96 (0.41; 2.26)	No studies
Education:Exercise	**9.57 (1.22; 75.20)**	No studies
Education:Exercise education	1.07 (0.57; 2.00)	No studies
Education:Exercise ergonomics	1.37 (0.23; 8.27)	No studies
Education:Usual care	0.91 (0.51; 1.64)	No studies
Education ergonomics:Exercise	**9.93 (1.25; 78.78)**	No studies
Education ergonomics:Exercise education	1.11 (0.57; 2.15)	1.09 (0.55; 2.14)
Education ergonomics:Exercise ergonomics	1.42 (0.23; 8.67)	1.42 (0.23; 8.70)
Education ergonomics:Usual care	0.95 (0.51; 1.76)	0.95 (0.51; 1.77)
Exercise:Exercise education	**0.11 (0.02; 0.82)**	No studies
Exercise:Exercise ergonomics	0.14 (0.01; 1.94)	No studies
Exercise:Usual care	**0.10 (0.01; 0.69)**	No studies
Exercise education:Exercise ergonomics	1.28 (0.23; 7.10)	1.31 (0.23; 7.31)
Exercise education:Usual care	0.85 (0.68; 1.07)	0.87 (0.67; 1.13)
Exercise ergonomics:Usual care	0.67 (0.12; 3.65)	0.67 (0.12; 3.66)

RR: risk ratio of NMA. CI: confident interval.

5.2 Number of days of sick leave caused by BP

Studies of high risk of bias of overall domains (Gundewall 1993, Kellett 1991, Soukup 1999, van Poppel 1998, Warming 2008) were excluded.

Comparison	All studies<br>RR (95% CI)	Excluding studies<br>RR (95% CI)
Education:Exercise	0.03 (-0.93; 1.00)	-0.12 (-1.37; 1.12)
Education:Exercise education	0.19 (-0.71; 1.09)	0.30 (-0.81; 1.42)
Education:Usual care	-0.20 (-1.09; 0.69)	-0.16 (-1.27; 0.96)
Exercise:Exercise education	0.16 (-0.57; 0.88)	0.43 (-0.68; 1.53)
Exercise:Usual care	-0.24 (-0.92; 0.44)	-0.04 (-1.14; 1.07)
Exercise education:Usual care	**-0.39 (-0.77; -0.02)**	-0.46 (-1.01; 0.09)

RR: risk ratio of NMA. CI: confident interval.

5.3 Number of people who had BP

Studies of high risk of bias of overall domains (Soukup 1999, van Poppel 1998, Warming 2008) were excluded.

Comparison	All studies<br>RR (95% CI)	Excluding studies<br>RR (95% CI)
Back belts:Education	0.93 (0.53; 1.63)	No studies
Back belts:Education ergonomics	0.97 (0.53; 1.78)	No studies
Back belts:Exercise education	1.30 (0.79; 2.13)	No studies
Back belts:Exercise ergonomics	0.52 (0.05; 5.75)	No studies
Back belts:Usual care	1.04 (0.66; 1.65)	No studies
Education:Education ergonomics	1.04 (0.63; 1.73)	No studies
Education:Exercise education	1.39 (0.98; 1.97)	No studies
Education:Exercise ergonomics	0.56 (0.05; 6.03)	No studies
Education:Usual care	1.12 (0.81; 1.53)	No studies
Education ergonomics:Exercise education	1.34 (0.87; 2.07)	1.27 (0.82; 1.96)
Education ergonomics:Exercise ergonomics	0.54 (0.05; 5.88)	0.54 (0.05; 5.87)
Education ergonomics:Usual care	1.07 (0.72; 1.60)	1.08 (0.73; 1.59)
Exercise education:Exercise ergonomics	0.40 (0.04; 4.27)	0.42 (0.04; 4.52)
Exercise education:Usual care	**0.80 (0.67; 0.96)**	0.85 (0.70; 1.03)
Exercise ergonomics:Usual care	2.00 (0.19; 21.16)	2.00 (0.17; 20.00)

RR: risk ratio of NMA. CI: confident interval.

Supplementary Materials S6: Comparison-Adjusted Funnel Plot

6.1 Number of people reporting a work absence due to BP

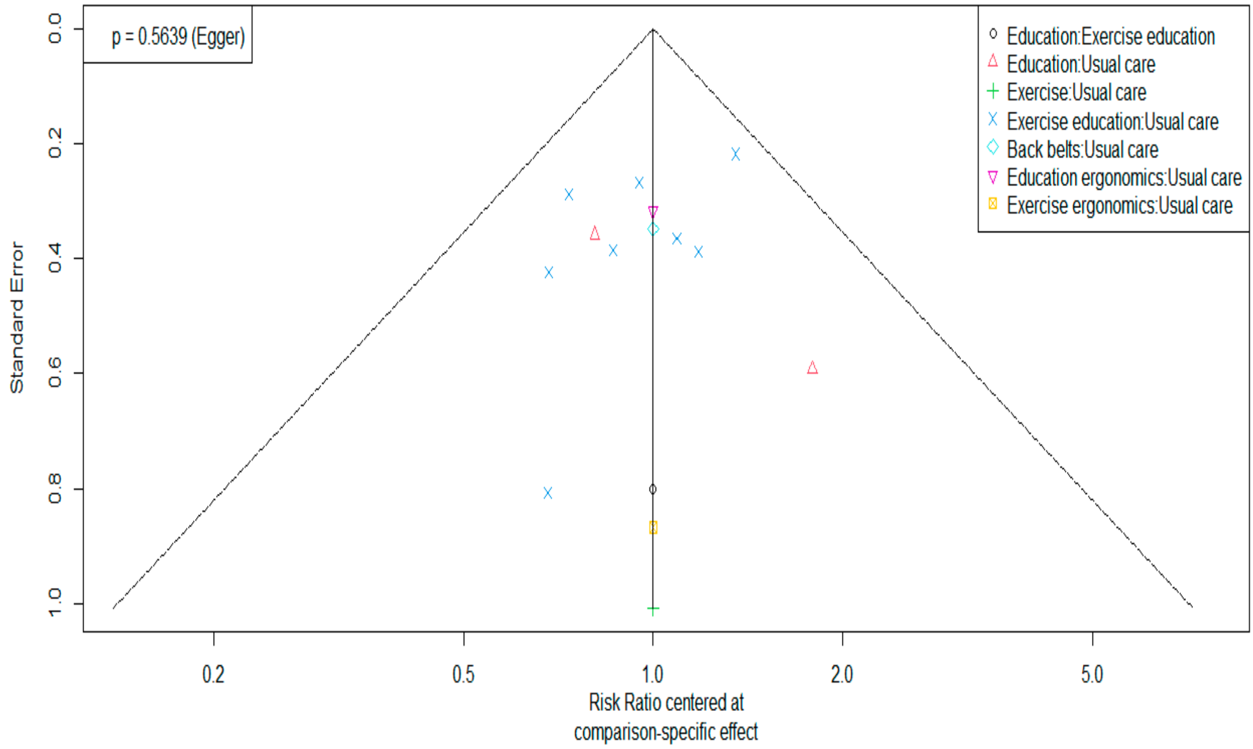

6.2 Number of people who had BP

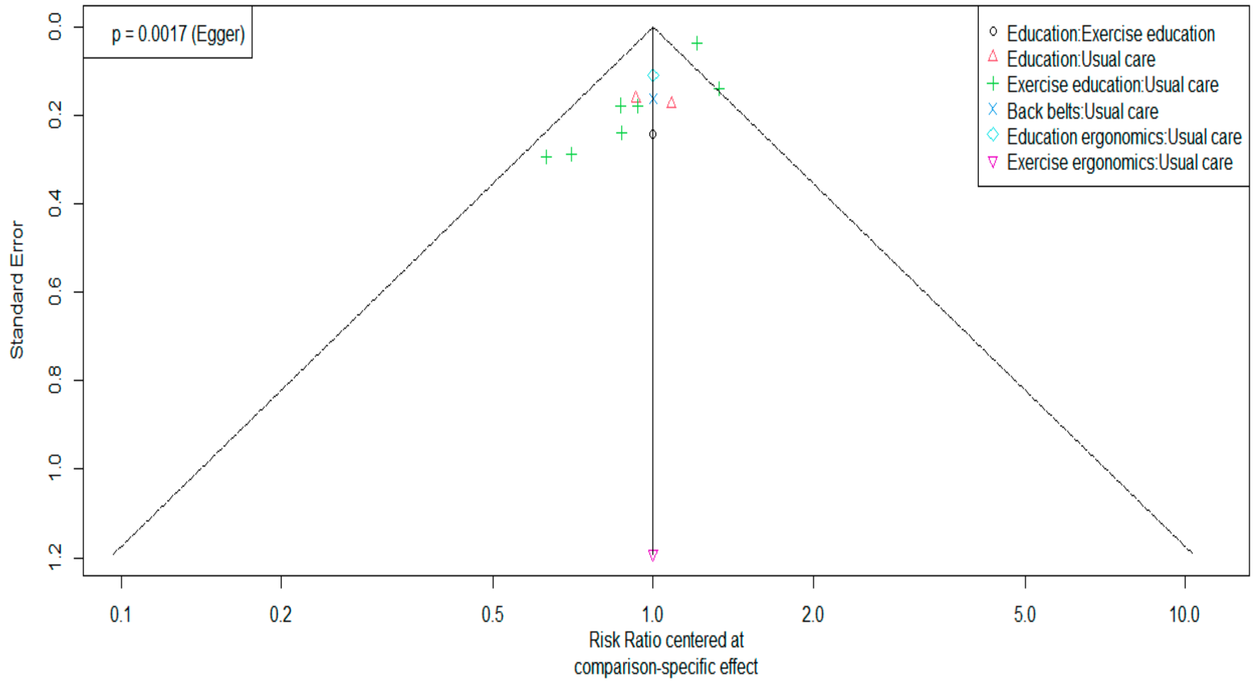

Supplementary Materials S7: Certainty of Direct Evidence Assessment

7.1 Number of people reporting a work absence due to BP

Comparison	No.	Risk of bias	Inconsistency	Indirectness	Imprecision	Publication bias	RR (95% CI)	Quality of evidence
Back belts:Usual care	1	Serious[3]	NA[2]	Not Serious	Serious[4]	Unclear[1]	1.44 (0.73; 2.86)	Low
Education:Exercise education	1	Serious[3]	NA[2]	Not Serious	Serious[4]	Unclear[1]	2.65 (0.55; 1.74)	Low
Education:Usual care	2	Serious[3]	Not Serious	Not Serious	Serious[4]	Unclear[1]	0.86 (0.47; 1.57)	Low
Education ergonomics:Usual care	1	Not Serious	NA[2]	Not Serious	Serious[4]	Unclear[1]	0.95 (0.51; 1.76)	Moderate
Exercise:Usual care	1	Not Serious	NA[2]	Not Serious	Serious[4]	Unclear[1]	0.10 (0.01; 0.69)	Moderate
Exercise education:Usual care	8	Not Serious	Not Serious	Not Serious	Not Serious	Unclear[1]	0.85 (0.68; 1.07)	High
Exercise ergonomics:Usual care	1	Not Serious	NA[2]	Not Serious	Serious[4]	Unclear[1]	0.67 (0.12; 3.65)	Moderate

[1] The funnel plot or Egger’s test was not performed because of <10 studies.

[2] Unable to assess because there are <2 studies available.

[3] Studies had a high risk of bias in the domain of overall risk of bias

[4] Serious because of <400 participants for this outcome

No.: Number of studies

7.2 Number of days of sick leave caused by BP

Comparison	No.	Risk of bias	Inconsistency	Indirectness	Imprecision	Publication bias	RR (95% CI)	Quality of evidence
Education:Exercise	1	Not Serious	NA[2]	Not Serious	Serious[4]	Unclear[1]	-0.12 (-1.15; 0.91)	High
Education:Exercise education	1	Not Serious	NA[2]	Not Serious	Serious[4]	Unclear[1]	0.14 (-0.89; 1.17)	Moderate
Education:Usual care	1	Serious[3]	NA[2]	Not Serious	Serious[4]	Unclear[1]	0.00 (-1.03; 1.03)	Low
Exercise:Exercise education	1	Not Serious	NA[2]	Not Serious	Serious[4]	Unclear[1]	0.26 (-0.75; 1.28)	Moderate
Exercise:Usual care	2	Not Serious	Not Serious	Not Serious	Serious[4]	Unclear[1]	-0.21 (-0.94; 0.51)	High
Exercise education:Usual care	7	Not Serious	Serious[5]	Not Serious	Not Serious	Unclear[1]	-0.37 (-0.75; 0.01)	Moderate

[1] The funnel plot or Egger's test was not performed because of <10 studies.

[2] Unable to assess because there are <2 studies available.

[3] Studies had a high risk of bias in the domain of overall risk of bias

[4] Serious because of <400 participants for this outcome

[5] Serious because large heterogeneity between studies I^2 >50%

No.: Number of studies

7.3 Number of people who had BP

Comparison	No.	Risk of bias	Inconsistency	Indirectness	Imprecision	Publication bias	RR (95% CI)	Quality of evidence
Back belts:Usual care	1	Serious[3]	NA[2]	Not Serious	Serious[4]	Unclear[1]	1.04 (0.66; 1.65)	Low
Education:Exercise education	1	Serious[3]	Not Serious	Not Serious	Serious[4]	Unclear[1]	1.67 (0.94; 2.97)	Low
Education:Usual care	2	Serious[3]	Not Serious	Not Serious	Serious[4]	Unclear[1]	1.08 (0.78; 1.51)	Low
Education ergonomics:Usual care	1	Not Serious	NA[2]	Not Serious	Serious[4]	Unclear[1]	1.07 (0.72; 1.60)	Moderate
Exercise education:Usual care	7	Not Serious	Not Serious	Not Serious	Serious[4]	Unclear[1]	0.81 (0.67; 0.97)	Moderate

Exercise ergonomics:Usual care	1	Not Serious	NA[2]	Not Serious	Serious[4]	Unclear[1]	2.00 (0.19; 21.16)	Moderate

[1] The funnel plot or Egger's test was not performed because of <10 studies.

[2] Unable to assess because there are <2 studies available.

[3] Studies had a high risk of bias in the domain of overall risk of bias

[4] Serious because of <400 participants for this outcome

No.: Number of studies

Supplementary Materials S8: Certainty of Network Evidence Assessment

8.1 Number of people reporting a work absence due to BP

Comparison	Direct Evidence		Indirect Evidence		Network Meta-Analysis	
	RR (95% CI)	Quality of evidence	RR (95% CI)	Quality of evidence	RR (95% CI)	Quality of evidence
Back belts:Education	-	-	1.58 (0.64; 3.90)	Low[3]	1.58 (0.64; 3.90)	Low
Back belts:Education ergonomics	-	-	1.53 (0.61; 3.84)	Low[3]	1.53 (0.61; 3.84)	Low
Back belts:Exercise	-	-	15.17 (1.87; 122.70)	Low[2,3]	15.17 (1.87; 122.70)	Low
Back belts:Exercise education	-	-	1.70 (0.83; 3.48)	Low[3]	1.70 (0.83; 3.48)	Low
Back belts:Exercise ergonomics	-	-	2.17 (0.35; 13.55)	Low[2,3]	2.17 (0.35; 13.55)	Low
Back belts:Usual care	1.44 (0.73; 2.86)	Low	-	-	1.44 (0.73; 2.86)	Low

Comparison	Direct Evidence		Indirect Evidence		Network Meta-Analysis	
	RR (95% CI)	Quality of evidence	RR (95% CI)	Quality of evidence	RR (95% CI)	Quality of evidence
Education:Education ergonomics	-	-	0.96 (0.41; 2.26)	Low[3]	0.96 (0.41; 2.26)	Low
Education:Exercise	-	-	9.57 (1.22; 75.20)	Low[2,3]	9.57 (1.22; 75.20)	Low
Education:Exercise education	2.65 (0.55; 1.74)	Low	0.90 (0.46; 1.78)	Low[3]	1.07 (0.57; 2.00)	Low
Education:Exercise ergonomics	-	-	1.37 (0.23; 8.27)	Low[2,3]	1.37 (0.23; 8.27)	Low
Education:Usual care	0.86 (0.47; 1.57)	Low	3.33 (0.19; 59.12)	Low[2,3]	0.91 (0.51; 1.64)	Low
Education ergonomics:Exercise	-	-	9.93 (1.25; 78.78)	Moderate[2,3]	9.93 (1.25; 78.78)	Moderate
Education ergonomics:Exercise education	-	-	1.11 (0.57; 2.15)	Moderate[3]	1.11 (0.57; 2.15)	Moderate
Education ergonomics:Exercise ergonomics	-	-	1.42 (0.23; 8.67)	Moderate[2,4]	1.42 (0.23; 8.67)	Moderate
Education ergonomics:Usual care	0.95 (0.51; 1.76)	Moderate	-	-	0.95 (0.51; 1.76)	Moderate
Exercise:Exercise education	-	-	0.11 (0.02; 0.82)	Moderate[3]	0.11 (0.02; 0.82)	Moderate
Exercise:Exercise ergonomics	-	-	0.14 (0.01; 1.94)	Moderate[3]	0.14 (0.01; 1.94)	Moderate
Exercise:Usual care	0.10 (0.01; 0.69)	Moderate	-	-	0.10 (0.01; 0.69)	Moderate

Comparison	Direct Evidence		Indirect Evidence		Network Meta-Analysis	
	RR (95% CI)	Quality of evidence	RR (95% CI)	Quality of evidence	RR (95% CI)	Quality of evidence
Exercise education:Exercise ergonomics	-	-	1.28 (0.23; 7.10)	Moderate[2,3]	1.28 (0.23; 7.10)	Moderate
Exercise education:Usual care	0.85 (0.68; 1.07)	High	0.03 (0.00; 3.36)	Low[3]	0.85 (0.68; 1.07)	High[1]
Exercise ergonomics:Usual care	0.67 (0.12; 3.65)	Moderate	-	-	0.67 (0.12; 3.65)	Moderate

[1] Choose the evidence that contributes the most

[2] Imprecise (wide 95% CI)

[3] Choose the lower rating of two direct comparisons

8.2 Number of days of sick leave caused by BP

Comparison	Direct Evidence		Indirect Evidence		Network Meta-Analysis	
	RR (95% CI)	Quality of evidence	RR (95% CI)	Quality of evidence	RR (95% CI)	Quality of evidence
Education:Exercise	-0.12 (-1.15; 0.91)	High	1.06 (-1.57; 3.70)	Low[3]	0.03 (-0.93; 1.00)	High[2]
Education:Exercise education	0.14 (-0.89; 1.17)	Moderate	0.35 (-1.51; 2.21)	Low[3]	0.19 (-0.71; 1.09)	Moderate[1]
Education:Usual care	0.00 (-1.03; 1.03)	Low	-0.82 (-2.62; 0.97)	Moderate[3]	-0.20 (-1.09; 0.69)	Low[1]
Exercise:Exercise education	0.26 (-0.75; 1.28)	Moderate	0.05 (-0.98; 1.07)	Moderate[3]	0.16 (-0.57; 0.88)	Moderate
Exercise:Usual care	-0.21 (-0.94; 0.51)	High	-0.42 (-2.38; 1.54)	No need[2]	-0.24 (-0.92; 0.44)	High[2]

Comparison	Direct Evidence		Indirect Evidence		Network Meta-Analysis	
	RR (95% CI)	Quality of evidence	RR (95% CI)	Quality of evidence	RR (95% CI)	Quality of evidence
Exercise education:Usual care	-0.37 (-0.75; 0.01)	Moderate	-1.54 (-4.29; 1.22)	Low[3]	-0.39 (-0.77; -0.02)	Moderate[1]

[1] Choose the evidence that contributes the most

[2] High certainty and the direct evidence contributes more than indirect evidence

[3] Choose the lower rating of two direct comparisons

8.3 Number of people who had BP

Comparison	Direct Evidence		Indirect Evidence		Network Meta-Analysis	
	RR (95% CI)	Quality of evidence	RR (95% CI)	Quality of evidence	RR (95% CI)	Quality of evidence
Back belts:Education	-	-	0.93 (0.53; 1.63)	Low[3]	0.93 (0.53; 1.63)	Low
Back belts:Education ergonomics	-	-	0.97 (0.53; 1.78)	Low[3]	0.97 (0.53; 1.78)	Low
Back belts:Exercise education	-	-	1.30 (0.79; 2.13)	Low[3]	1.30 (0.79; 2.13)	Low
Back belts:Exercise ergonomics	-	-	0.52 (0.05; 5.75)	Low[2,3]	0.52 (0.05; 5.75)	Low
Back belts:Usual care	1.04 (0.66; 1.65)	Low	-	-	1.04 (0.66; 1.65)	Low
Education:Education ergonomics	-	-	1.04 (0.63; 1.73)	Low[3]	1.04 (0.63; 1.73)	Low

Comparison	Direct Evidence		Indirect Evidence		Network Meta-Analysis	
	RR (95% CI)	Quality of evidence	RR (95% CI)	Quality of evidence	RR (95% CI)	Quality of evidence
Education:Exercise education	1.67 (0.94; 2.97)	Low	1.26 (0.81; 1.95)	Low[3]	1.39 (0.98; 1.97)	Low
Education:Exercise ergonomics	-	-	0.56 (0.05; 6.03)	Low[2, 3]	0.56 (0.05; 6.03)	Low
Education:Usual care	1.08 (0.78; 1.51)	Low	1.59 (0.52; 4.88)	Low[3]	1.12 (0.81; 1.53)	Low
Education ergonomics:Exercise education	-	-	1.34 (0.87; 2.07)	Moderate[3]	1.34 (0.87; 2.07)	Moderate
Education ergonomics:Exercise ergonomics	-	-	0.54 (0.05; 5.88)	Moderate[2,3]	0.54 (0.05; 5.88)	Moderate
Education ergonomics:Usual care	1.07 (0.72; 1.60)	Moderate	-	-	1.07 (0.72; 1.60)	Moderate
Exercise education:Exercise ergonomics	-	-	0.40 (0.04; 4.27)	Moderate[3]	0.40 (0.04; 4.27)	Moderate
Exercise education:Usual care	0.81 (0.67; 0.97)	Moderate	0.45 (0.07; 2.90)	No need[1]	0.80 (0.67; 0.96)	Moderate[1]
Exercise ergonomics:Usual care	2.00 (0.19; 21.16)	Moderate	-	-	2.00 (0.19; 21.16)	Moderate

[1] High certainty and the direct evidence contributes more than indirect evidence

[2] Imprecise (wide 95% CI)

[3] Choose the lowest of the two direct ratings

<u>Supplementary Materials S9: Subgroup analysis</u>

9.1 Number of people reporting a work absence due to BP

**Resistance (P-score = 0.969)**	.	.	.	.	*0.10 (0.01;0.69)*	.	.
0.13 (0.02; 0.95)	**Resistance stretching education (P-score = 0.638)**	.	.	.	*0.74 (0.55;0.99)*	.	.
0.16 (0.01; 2.05)	1.27 (0.25; 6.33)	**Aerobic resistance education (P-score = 0.629)**	.	.	0.58 (0.12;2.84)	.	0.38 (0.08;1.81)
0.14 (0.01; 1.94)	1.11 (0.20; 6.22)	0.87 (0.09; 8.93)	**Motor control ergonomics (P-score = 0.563)**	.	0.67 (0.12;3.65)	.	.
0.09 (0.01; 0.78)	0.73 (0.32; 1.64)	0.57 (0.10; 3.33)	0.66 (0.10; 4.24)	**Resistance education (P-score = 0.381)**	1.01 (0.47;2.17)	.	.
0.10 (0.01; 0.69)	*0.74 (0.55; 0.99)*	0.58 (0.12; 2.84)	0.67 (0.12; 3.65)	1.01 (0.47; 2.17)	**Usual care (P-score = 0.376)**	0.86 (0.56;1.32)	0.65 (0.20;2.06)
0.08 (0.01; 0.62)	0.64 (0.38; 1.07)	0.50 (0.10; 2.59)	0.57 (0.10; 3.32)	0.87 (0.37; 2.09)	0.86 (0.56; 1.32)	**Stretching education (P-score = 0.261)**	.
0.06 (0.01; 0.61)	0.48 (0.14; 1.58)	0.38 (0.08; 1.81)	0.43 (0.06; 3.38)	0.66 (0.16; 2.62)	0.65 (0.20; 2.06)	0.75 (0.22; 2.58)	**Education (P-score = 0.182)**

9.2 Number of days of sick leave caused by BP

**Resistance stretching Education (P-score = 0.78)**	.	-0.14 (-1.14; 0.86)	-0.26 (-1.25; 0.72)	-0.47 (-0.87; -0.07)	.
0.10 (-0.98; 1.18)	**Resistance (P-score = 0.76)**	.	.	-0.57 (-1.57; 0.44)	.
-0.31 (-1.20; 0.58)	-0.40 (-1.75; 0.94)	**Education (P-score = 0.49)**	-0.12 (-1.12; 0.87)	0.00 (-0.99; 0.99)	.
-0.43 (-1.30; 0.44)	-0.53 (-1.86; 0.80)	-0.12 (-1.12; 0.87)	**Resistance stretching (P-score = 0.39)**	0.12 (-0.85; 1.10)	.
-0.47 (-0.87; -0.07)	-0.57 (-1.57; 0.44)	-0.16 (-1.05; 0.73)	-0.04 (-0.91; 0.83)	**Usual care (P-score = 0.31)**	-0.12 (-1.01; 0.77)
-0.59 (-1.57; 0.39)	-0.69 (-2.03; 0.65)	-0.28 (-1.54; 0.98)	-0.16 (-1.41; 1.08)	-0.12 (-1.01; 0.77)	**Stretching education (P-score = 0.28)**

9.3 Number of people who had BP

**Resistance stretching Education (P-score = 0.88)**	.	.	*0.67 (0.54;0.82)*	.	.
0.95 (0.57;1.59)	**Aerobic resistance Education (P-score = 0.80)**	.	0.70 (0.44;1.13)	.	.
0.69 (0.55;0.86)	0.72 (0.45;1.16)	**Stretching education (P-score = 0.47)**	0.97 (0.91;1.04)	.	.
0.67 (0.54;0.82)	0.70 (0.44;1.13)	0.97 (0.91;1.04)	**Usual care (P-score = 0.34)**	0.93 (0.71;1.23)	0.50 (0.05;5.17)
0.62 (0.44;0.88)	0.66 (0.38;1.13)	0.91 (0.69;1.20)	0.93 (0.71;1.23)	**Resistance education (P-score = 0.27)**	.
0.33 (0.03;3.49)	0.35 (0.03;3.81)	0.49 (0.05;5.03)	0.50 (0.05;5.17)	0.54 (0.05;5.62)	**Motor control Ergonomics (P-score = 0.25)**

Note:

Aerobic resistance education = Aerobic exercise and resistance exercise combined with education

Resistance stretching education = Resistance exercise and stretching exercise combined with education

Motor control ergonomics = Motor control exercise and ergonomic adjustments

Resistance education = Resistance exercise combined with education

Stretching education = Stretching exercise combined with education

Resistance stretching = Resistance exercise and stretching exercise

Supplementary Materials S10: Summary of data

10.1 Number of people reporting a work absence due to BP

First author, Year	Number of events	Number of participants	Type of intervention
Lonn 1999	7	38	Exercise education
Lonn 1999	11	35	Usual care
Glomsrod 2001	12	37	Exercise education
Glomsrod 2001	18	35	Usual care
Gundewall 1993	1	28	Exercise
Gundewall 1993	12	32	Usual care
Ijzelenberg 2007	18	185	Education ergonomics
Ijzelenberg 2007	18	175	Usual care
Kellett 1991	8	37	Exercise education
Kellett 1991	14	48	Usual care
Soukup 1999	10	34	Exercise education
Soukup 1999	11	35	Usual care
Soukup 2001	13	31	Exercise education
Soukup 2001	18	35	Usual care
van Poppel 1998	12	142	Education
van Poppel 1998	17	140	Usual care
van Poppel 1998	17	134	Back belts
van Poppel 1998	13	148	Usual care
Warming 2008	2	35	Exercise education
Warming 2008	5	33	Education
Warming 2008	5	51	Usual care
Roussel 2015	2	25	Exercise ergonomics
Roussel 2015	3	25	Usual care
Chaleat-Valayer 2016	35	147	Exercise education
Chaleat-Valayer 2016	31	151	Usual care
Ferreira 2021	11	51	Exercise education
Ferreira 2021	10	47	Usual care

10.2 Number of days of sick leave caused by BP

First author, Year	Mean	SD*	Number of participants	Type of intervention
Lonn 1999	10.4	9.3	38	Exercise education
Lonn 1999	37.8	28	35	Usual care
Glomsrod 2001	14.4	12.7	37	Exercise education
Glomsrod 2001	63.9	76.3	35	Usual care
Gundewall 1993	1	0.189	28	Exercise
Gundewall 1993	4.84	9.26	32	Usual care
Kellett 1991	2.73	7.63	37	Exercise education
Kellett 1991	4.13	9.32	48	Usual care
Soukup 1999	29.9	55.2	34	Exercise education
Soukup 1999	37.8	28	35	Usual care
Soukup 2001	52.4	97.9	31	Exercise education
Soukup 2001	63.9	76.3	35	Usual care
van Poppel 1998	0.5	0	142	Education
van Poppel 1998	0.3	0	140	Usual care
van Poppel 1998	0.4	0	134	Back belts
van Poppel 1998	0.4	0	148	Usual care
Chaleat-Valayer 2016	32	65	147	Exercise education
Chaleat-Valayer 2016	25	50	151	Usual care
Suni 2018	0.15	0.96	34	Exercise education
Suni 2018	4.17	26.89	35	Exercise
Suni 2018	2.3	4.04	27	Education
Suni 2018	2.29	11.92	38	Usual care

*SD: Standard Deviation

10.3 Number of people who had BP

First author, Year	Number of events	Number of participants	Type of intervention
Lonn 1999	11	38	Exercise education
Lonn 1999	20	35	Usual care
Glomsrod 2001	20	37	Exercise education
Glomsrod 2001	27	35	Usual care
Ijzelenberg 2007	92	185	Education ergonomics
Ijzelenberg 2007	81	175	Usual care
Soukup 1999	11	34	Exercise education
Soukup 1999	20	35	Usual care
Soukup 2001	18	31	Exercise education
Soukup 2001	27	35	Usual care
van Poppel 1998	50	142	Education
van Poppel 1998	49	140	Usual care
van Poppel 1998	48	134	Back belts
van Poppel 1998	51	148	Usual care
Warming 2008	14	35	Exercise education
Warming 2008	22	33	Education
Warming 2008	29	51	Usual care
Roussel 2015	2	25	Exercise ergonomics
Roussel 2015	1	25	Usual care
Chaleat-Valayer 2016	121	133	Exercise education
Chaleat-Valayer 2016	129	138	Usual care
Ferreira 2021	36	51	Exercise education
Ferreira 2021	31	47	Usual care

Supplementary Materials S11: PRISMA NMA Checklist of Items to Include When Reporting A Systematic Review Involving a Network Meta-analysis

Section/Topic	Item #	Checklist Item	Reported on Page #
**TITLE**			
Title	1	Identify the report as a systematic review *incorporating a network meta-analysis (or related form of meta-analysis).*	# 1
**ABSTRACT**			
Structured summary	2	Provide a structured summary including, as applicable:	
		**Background:** main objectives	# 1
		**Methods:** data sources; study eligibility criteria, participants, and interventions; study appraisal; and *synthesis methods, such as network meta-analysis.*	# 1
		**Results:** number of studies and participants identified; summary estimates with corresponding confidence/credible intervals; *treatment rankings may also be discussed. Authors may choose to summarize pairwise comparisons against a chosen treatment included in their analyses for brevity.*	# 1
		**Discussion/Conclusions:** limitations; conclusions and implications of findings.	# 1
		**Other:** primary source of funding; systematic review registration number with registry name.	N/A
**INTRODUCTION**			
Rationale	3	Describe the rationale for the review in the context of what is already known*, including mention of why a network meta-analysis has been conducted.*	# 1,2
Objectives	4	Provide an explicit statement of questions being addressed, with reference to participants, interventions, comparisons, outcomes, and study design (PICOS).	# 1,2
**METHODS**			
Protocol and registration	5	Indicate whether a review protocol exists and if and where it can be accessed (e.g., Web address); and, if available, provide registration information, including registration number.	# 2
Eligibility criteria	6	Specify study characteristics (e.g., PICOS, length of follow-up) and report characteristics (e.g., years considered, language, publication status) used as criteria for eligibility, giving rationale. *Clearly describe eligible treatments included in the treatment network, and note whether any have been clustered or merged into the same node (with justification).*	# 2

Information sources	7	Describe all information sources (e.g., databases with dates of coverage, contact with study authors to identify additional studies) in the search and date last searched.	# 2
Search	8	Present full electronic search strategy for at least one database, including any limits used, such that it could be repeated.	# 2
Study selection	9	State the process for selecting studies (i.e., screening, eligibility, included in systematic review, and, if applicable, included in the meta-analysis).	Figure 1
Data collection process	10	Describe method of data extraction from reports (e.g., piloted forms, independently, in duplicate) and any processes for obtaining and confirming data from investigators.	# 2
Data items	11	List and define all variables for which data were sought (e.g., PICOS, funding sources) and any assumptions and simplifications made.	# 3
**Geometry of the network**	**S1**	Describe methods used to explore the geometry of the treatment network under study and potential biases related to it. This should include how the evidence base has been graphically summarized for presentation, and what characteristics were compiled and used to describe the evidence base to readers.	Figure 2
Risk of bias within individual studies	12	Describe methods used for assessing risk of bias of individual studies (including specification of whether this was done at the study or outcome level), and how this information is to be used in any data synthesis.	# 3
Summary measures	13	State the principal summary measures (e.g., risk ratio, difference in means). *Also describe the use of additional summary measures assessed, such as treatment rankings and surface under the cumulative ranking curve (SUCRA) values, as well as modified approaches used to present summary findings from meta-analyses.*	# 3
Planned methods of analysis	14	Describe the methods of handling data and combining results of studies for each network meta-analysis. This should include, but not be limited to:<br>• *Handling of multi-arm trials;*<br>• *Selection of variance structure;*<br>• *Selection of prior distributions in Bayesian analyses; and*<br>• *Assessment of model fit.*	# 3
**Assessment of Inconsistency**	**S2**	Describe the statistical methods used to evaluate the agreement of direct and indirect evidence in the treatment network(s) studied. Describe efforts taken to address its presence when found.	# 3
Risk of bias across studies	15	Specify any assessment of risk of bias that may affect the cumulative evidence (e.g., publication bias, selective reporting within studies).	# 3

Additional analyses	16	Describe methods of additional analyses if done, indicating which were pre-specified. This may include, but not be limited to, the following:<br>• Sensitivity or subgroup analyses;<br>• Meta-regression analyses;<br>• *Alternative formulations of the treatment network; and*<br>• *Use of alternative prior distributions for Bayesian analyses (if applicable).*	# 3
**RESULTS†**			
Study selection	17	Give numbers of studies screened, assessed for eligibility, and included in the review, with reasons for exclusions at each stage, ideally with a flow diagram.	Figure 1
**Presentation of network structure**	**S3**	Provide a network graph of the included studies to enable visualization of the geometry of the treatment network.	Figure 2
**Summary of network geometry**	**S4**	Provide a brief overview of characteristics of the treatment network. This may include commentary on the abundance of trials and randomized patients for the different interventions and pairwise comparisons in the network, gaps of evidence in the treatment network, and potential biases reflected by the network structure.	# 7
Study characteristics	18	For each study, present characteristics for which data were extracted (e.g., study size, PICOS, follow-up period) and provide the citations.	Table 1
Risk of bias within studies	19	Present data on risk of bias of each study and, if available, any outcome level assessment.	Supplementary Materials S2
Results of individual studies	20	For all outcomes considered (benefits or harms), present, for each study: 1) simple summary data for each intervention group, and 2) effect estimates and confidence intervals. *Modified approaches may be needed to deal with information from larger networks.*	Supplementary Materials S10
Synthesis of results	21	Present results of each meta-analysis done, including confidence/credible intervals. *In larger networks, authors may focus on comparisons versus a particular comparator (e.g. placebo or standard care), with full findings presented in an appendix. League tables and forest plots may be considered to summarize pairwise comparisons.* If additional summary measures were explored (such as treatment rankings), these should also be presented.	Table 2
**Exploration for inconsistency**	**S5**	Describe results from investigations of inconsistency. This may include such information as measures of model fit to compare consistency and inconsistency models, *P* values from statistical tests, or summary of inconsistency estimates from different parts of the treatment network.	# 10

Risk of bias across studies	22	Present results of any assessment of risk of bias across studies for the evidence base being studied.	Supplementary Materials S3
Results of additional analyses	23	Give results of additional analyses, if done (e.g., sensitivity or subgroup analyses, meta-regression analyses*, alternative network geometries studied, alternative choice of prior distributions for Bayesian analyses,* and so forth).	# 10
**DISCUSSION**			
Summary of evidence	24	Summarize the main findings, including the strength of evidence for each main outcome; consider their relevance to key groups (e.g., healthcare providers, users, and policy-makers).	# 11
Limitations	25	Discuss limitations at study and outcome level (e.g., risk of bias), and at review level (e.g., incomplete retrieval of identified research, reporting bias). *Comment on the validity of the assumptions, such as transitivity and consistency. Comment on any concerns regarding network geometry (e.g., avoidance of certain comparisons).*	# 11
Conclusions	26	Provide a general interpretation of the results in the context of other evidence, and implications for future research.	# 12
**FUNDING**			
Funding	27	Describe sources of funding for the systematic review and other support (e.g., supply of data); role of funders for the systematic review. This should also include information regarding whether funding has been received from manufacturers of treatments in the network and/or whether some of the authors are content experts with professional conflicts of interest that could affect use of treatments in the network.	# 12

PICOS = population, intervention, comparators, outcomes, study design.
* Text in italics indicateS wording specific to reporting of network meta-analyses that has been added to guidance from the PRISMA statement.
† Authors may wish to plan for use of appendices to present all relevant information in full detail for items in this section.
